# The Early-Onset Myocardial Infarction Associated PHACTR1 Gene Regulates Skeletal and Cardiac Alpha-Actin Gene Expression

**DOI:** 10.1371/journal.pone.0130502

**Published:** 2015-06-22

**Authors:** Annina Kelloniemi, Zoltan Szabo, Raisa Serpi, Juha Näpänkangas, Pauli Ohukainen, Olli Tenhunen, Leena Kaikkonen, Elina Koivisto, Zsolt Bagyura, Risto Kerkelä, Margret Leosdottir, Thomas Hedner, Olle Melander, Heikki Ruskoaho, Jaana Rysä

**Affiliations:** 1 Institute of Biomedicine, Department of Pharmacology and Toxicology, University of Oulu, Oulu, Finland; 2 Department of Pathology, Oulu University Hospital, University of Oulu, Oulu, Finland; 3 Heart Center, Semmelweis University, Budapest, Hungary; 4 Medical Research Center Oulu, Oulu University Hospital, University of Oulu, Oulu, Finland; 5 Lund University, Department of Clinical Sciences, Malmö, Sweden; 6 Institute of Medicine, The Sahlgrenska Academy, University of Gothenburg, Gothenburg, Sweden; Loyola University Chicago, UNITED STATES

## Abstract

The phosphatase and actin regulator 1 (PHACTR1) locus is a very commonly identified hit in genome-wide association studies investigating coronary artery disease and myocardial infarction (MI). However, the function of PHACTR1 in the heart is still unknown. We characterized the mechanisms regulating Phactr1 expression in the heart, used adenoviral gene delivery to investigate the effects of Phactr1 on cardiac function, and analyzed the relationship between MI associated PHACTR1 allele and cardiac function in human subjects. Phactr1 mRNA and protein levels were markedly reduced (60%, *P*<0.01 and 90%, *P*<0.001, respectively) at 1 day after MI in rats. When the direct myocardial effects of Phactr1 were studied, the skeletal α-actin to cardiac α-actin isoform ratio was significantly higher (1.5-fold, *P*<0.05) at 3 days but 40% lower (*P*<0.05) at 2 weeks after adenovirus-mediated Phactr1 gene delivery into the anterior wall of the left ventricle. Similarly, the skeletal α-actin to cardiac α-actin ratio was lower at 2 weeks in infarcted hearts overexpressing Phactr1. In cultured neonatal cardiac myocytes, adenovirus-mediated Phactr1 overexpression for 48 hours markedly increased the skeletal α-actin to cardiac α-actin ratio, this being associated with an enhanced DNA binding activity of serum response factor. Phactr1 overexpression exerted no major effects on the expression of other cardiac genes or LV structure and function in normal and infarcted hearts during 2 weeks’ follow-up period. In human subjects, MI associated PHACTR1 allele was not associated significantly with cardiac function (n = 1550). Phactr1 seems to regulate the skeletal to cardiac α-actin isoform ratio.

## Introduction

Heart failure (HF) is one of the most common cardiovascular diseases with high morbidity and mortality in the Western world [1]. Coronary artery disease and its main complication, myocardial infarction (MI) are the main causes of adverse left ventricular (LV) remodelling and heart failure [2,3]. One fundamental feature of the hemodynamically stressed, failing heart is the appearance of the fetal gene program [4]. The immediate early genetic response to mechanical loading or neurohumoral hypertrophic stimuli in cardiac myocytes includes transcription of many genes such as c-*fos*, c-*jun* and early growth response -1 (EGR-1) [4–6]. Later, there is a reprogramming of cardiac gene expression e.g.up-regulation of atrial and brain natriuretic peptides (ANP and BNP) [7], and a switch to fetal isoforms of contractile proteins such as cardiac α-actin being substituted by skeletal α-actin and α-myosin heavy chain by β-myosin heavy chain [4,8]. Normally, the genes coding for skeletal α-actin and β-myosin heavy chain are silent in the adult heart but their expression is reactivated in response to pathological stress [4,6,9]. Thus, an analysis of the mechanisms that regulate the expression of fetal cardiac genes can provide new insights into the development of cardiac hypertrophy and heart failure.

Phosphatase and actin regulator 1 (PHACTR1) locus is one of the most commonly identified hits in genome-wide association studies (GWAS) for coronary artery disease and MI [10–14], but its physiological function in the heart is still unknown. Phactrs are a family of four phosphatase and actin regulatory proteins that function as modulators of protein phosphatase 1 (PP1) and bind to actin. Phactrs are expressed abundantly in the central nervous system and at lower levels in lung, heart, kidney and testis [15]. It is known that PHACTR1 regulates tubulogenesis and cell survival in human endothelial cells [16]. In melanoma and breast cancer cells Phactr1 is involved in actin dynamics, cell motility and invasive behaviour [17,18].

Here we have analyzed Phactr1 expression in the heart and used adenoviral gene delivery to investigate the effects of Phactr1 on cardiac function. Phactr1 was overexpressed by using adenovirus-mediated gene delivery in normal rat hearts and in hearts after experimental MI. Moreover we analyzed the mechanisms regulating Phactr1 expression and the effects of Phactr1 overexpression in cultured neonatal rat ventricular myocytes (NRVMs). These studies revealed that Phactr1 selectively skews the skeletal α-actin to cardiac α-actin isoform ratio both *in vivo* and *in vitro*.

## Methods

### Recombinant adenoviral vectors

The recombinant adenoviruses over-expressing the full length coding region of rat Phactr1 (GeneBank accession number BC098634) or cytoLacZ were generated by Viraquest (North Liberty, IA, USA).

### Animals

Male 2-3-month-old Sprague-Dawley rats weighting 250–350 g from the colony of the Center of Experimental Animals at the University of Oulu, Finland, were used. All rats were kept in individual plastic cages with free access to tap water and normal rat chow. A 12-h light and 12-h dark environmental light cycle was maintained. All experimental protocols were approved by the Animal Use and Care Committee of the University of Oulu and the Provincial Government of Western Finland Department of Social Affairs and Health. The studies conform to the *Guide for the Care and Use of Laboratory Animals* published by the US National Institutes of Health.

### Intramyocardial Gene Transfer

Local injection of adenoviral constructs into the left ventricular free wall is an efficient site-specific method of gene delivery that targets high expression of the transgene in the LV without affecting other organs or other regions of the heart [19,20]. Rats were anesthetized with medetomidine hydrochloride (Domitor, 250 μg/kg i.p.) and ketamine hydrochloride (Ketamine, 50 mg/kg i.p.), and then connected to the respirator through a tracheotomy. A left thoracotomy and pericardial incision was performed. Different doses of adenoviral constructs were first tested to increase LV Phactr1 protein levels by injecting recombinant adenovirus in a 100 μl volume using a Hamilton precision syringe directly into the anterior wall of the LV. The syringe was inserted in one site of the LV free wall (apex to base), and then slowly the solution was injected while withdrawing the syringe. After the procedure, anaesthesia was partially antagonized with atipamezole hydrochloride (Antisedan, 1.5 mg/kg i.p.) and rats were hydrated with physiological saline solution (5 ml s.c.). For postoperative analgesia, buprenorphine hydrochloride (Temgesic, 0.05–0.2 mg/kg s.c.) was administered. A total of 262 animals were used in these experiments.

### Myocardial infarction and adenoviral gene transfer *in vivo*

MI was produced by ligation of the left anterior descending coronary artery (LAD). The sham-operated rats underwent the same surgical procedure without the ligation of LAD. Recombinant adenovirus (5×10^8^ pfu) was injected into the anterior wall of the LV before the ligation of LAD as previously described [20]. The adenoviral gene delivery to the sham-operated hearts was performed using the same technique without the ligation of LAD.

### Echocardiographic measurements

Transthoracic echocardiography was performed using the Acuson Ultrasound System (SequoiaTM512) and a 15 MHz linear transducer (15L8) (Acuson, MountainView, CA, USA) as previously described [19,20]. Rats were anesthetized with ketamine (50 mg/kg i.p.) and xylazine (10 mg/kg i.p.). Using two-dimensional imaging, a short axis view of the left ventricle at the level of the papillary muscles was obtained, and a two dimensionally guided M-mode recording through the anterior and posterior walls of the LV was acquired. LV end-systolic and end-diastolic dimensions as well as the thickness of the interventricular septum and posterior wall were measured from the M-mode tracings. LV fractional shortening (FS) and ejection fraction (EF) were calculated from the M-mode LV dimensions using the following equations: FS(%) = {(LVEDD-LVESD)/LVEDD}×100 and EF(%) = {(LVEDD)^3^-(LVESD)^3^/LVEDD^3^}×100. An average of three measurements of each variable was used. Echocardiographic measurements were performed by a skilled sonographer (Z.S.) blinded to the treatments. After echocardiography, the animals were sacrificed, hearts were weighed and the tissue samples were immersed in liquid nitrogen and stored at -70°C for later analysis.

### Myocyte isolation and cell culture

Cell cultures of cardiac ventricular cells were isolated from 2-to-4 day old Sprague-Dawley-rats [21]. The animals were killed by cervical dislocation and the ventricles were excised and cut into small pieces and thereafter incubated 1–2 h at +37°C in a solution containing 100 mM NaCl, 10 mM KCl, 1.2 mM KH_2_PO_4_, 4.0 mM MgSO_4_, 50 mM taurine, 20 mM glucose, 10 mM Hepes, 2 mg/ml collagenase type 2 (Worthington, Lakewood, NJ, USA), 2 mg/ml pancreatin (Sigma-Aldrich, St Louis, MO, USA) and 1% penicillin–streptomycin (PS). After incubation the detached cells were collected in 15 ml Falcon tubes and centrifuged 900 rpm for 5 min. The supernatant and the top layer of the pellet containing damaged cells were discarded and the isolated cardiomyocytes were plated on 35 mm dishes at a density of 1.4 × 10^5^/cm^2^. The cells were cultured overnight in Dulbecco's modified Eagle's medium nutrient mixture F-12 ham with L-glutamine (DMEM/F12) containing 10% fetal bovine serum (FBS) and 1% PS. Thereafter the cells were cultured in complete serum free medium (CSFM) containing DMEM/F12, 2.5 mg/ml bovine serum albumin, 1 μM insulin, 5.64 μg/ml transferrin, 32 nM selenium, 2.8 mM Na-pyruvate, 0.1 nM T_3_ and 1% PS. The cells were infected with adenoviruses 24 hours after the cells had been plated at virus concentrations of 1, 2 or 4 MOI (multiplicity of infection). Fresh CSFM was changed 24 hours after the infection and cells were cultured for72 h before being washed twice with ice-cold PBS and stored at -70°C. Cyclic mechanical stretch was performed as described before [22]. Adenoviruses and the protocol to study influence of p38 and p38 MAPK isoforms in cultured NRVMs were performed as previously described [23]. Briefly, adenoviruses were added to the culture medium 18–24 h after the cells were plated and incubated for 24 h, at virus concentrations of 1–4 MOI (depending on the demands of the experiment).

### Isolation of RNA and quantitative RT-PCR analysis

Total RNA from left ventricular tissues was isolated by the guanidine thiocyanate-CsCl method as described previously [24] and total RNA from cultured myocytes was isolated with TRIzol Reagent according to the manufacturer’s protocol (Invitrogen, CA, USA) by using Phase Lock Gel system (Qiagen, Venlo, Netherlands).

RNA was analyzed by the real-time quantitative polymerase chain reaction (RT-PCR) with TaqMan chemistry on an ABI 7300 sequence detection system (Applied Biosystems, CA, USA) as previously described [24]. Primer and probe sequences for RNA detection are presented in Table 1. The results were normalized to 18S RNA quantified from the same samples.

**Table 1:** Sequences of primers and fluorogenic probes used in real time quantitative RT-qPCR analysis (sequences 5’ to 3’).

Gene	Sense Primer (Forward)	Antisense Primer (Reverse)	Fluorogenic Probe
ANP	GAAAAGCAAACTGAGGGCTCTG	CCTACCCCCGAAGCAGCT	TCGCTGGCCCTCGGAGCCT
BNP	TGGGCAGAAGATAGACCGGA	ACAACCTCAGCCCGTCACAG	CGGCGCAGTCAGTCGCTTGG
BMP-2	ACACCGTGCTCAGCTTCCAT	GTCGGGAAGTTTTCCCACTCA	ACGAAGAAGCCATCGAGGAACTTTCAGAA
caα-A	GGGCCCTCCATTGTCCA	GCACAATACTGTCGTCCTGAGTG	CGCAAGTGCTTCTGAGGCGGCTAC
c-fos	GGCTGAACCCTTTGATGACTTC	GGGCAGTCTCCGAGCCA	TGTTTCCGGCATCATCTAGGC
corin	CCCAGTGGACATATCTGTGGC	TTCAAAGCAATGGGCAACTGT	TGTCCTCATTGCCAAGAAGTGGGTCC
ET-1	ATGGACAAGGAGTGTGTCTACTTCTG	GGGACGACGCGCTCG	CACCTGGACATCATCTGGGTCAACACTC
GATA4	AAGGCTATGCATCTCCTGTCACT	CCAGGCTGTTCCAAGAGTCC	ACATCGCAGGCCAGCTCCAAGC
α-MHC	GCAGAAAATGCACGATGAGGA	CATTCATATTTATTGTGGGATAGCAAC	TAACCTGTCCAGCAGAAAGAGCCTCGC
β-MHC	GCTACCCAACCCTAAGGATGC	TCTGCCTAAGGTGCTGTTTCAA	TGTGAAGCCCTGAGACCTGGAGCC
Phactr1	CCCCGTGAAATTGCCTTGT	CAGATCAGGACTTTCTTTGGAGGTA	TGCCAGTGAAACTGTCGCCTCCG
PLB	AAGTCTGTCGCCACCGCA	TGGTGGAGGGCCAGGTT	CCTGCACCATGCCAACGCAGC
SERCA2a	CAGCCATGGAGAACGCTCA	CGTTGACGCCGAAGTGG	ACAAAGACCGTGGAGGAGGTGCTGG
skα-A	TCCTCCGCCGTTGGCT	AATCTATGTACACGTCAAAAACAGGC	CATCGCCGCCACTGCAGCC
α-SMA	TCCTGACCCTGAAGTATCCGATA	GGTGCCAGATCTTTTCCATGTC	AACACGGCATCATCACCAACTGGGA
18S	TGGTTGCAAAGCTGAAACTTAAAG	AGTCAAATTAAGCCGCAGGC	CCTGGTGGTGCCCTTCCGTCA

ANP, atrial natriuretic peptide; BNP, B-type natriuretic peptide; BMP-2, bone morphogenetic protein 2; caα-A, cardiac α-actin; ET-1, endothelin-1; α-MHC, α-myosin heavy chain; β-MHC, β-myosin heavy chain; Phactr1, Phosphatase and actin regulator 1; PLB, phopholamban; Serca2a, Sarcoplasmic reticulum Ca^2+^ ATPase 2a; skα-A, skeletal α-actin; α-SMA, α-smooth muscle actin; RT-PCR, real time quantitative reverse transcription-PCR.

### Protein extraction from tissue samples

The LV tissue samples were disintegrated in liquid nitrogen and homogenized in lysis buffer consisting of 20 mmol/l Tris–HCl (pH 7.5), 10 mM NaCl, 0.1 mM EDTA, 0.1 mM EGTA, 1 mM β-glycerophosphate, 1 mM Na_3_VO_4_, 2 mM benzamidine, 1 mM phenylmethylsulfoxide (PMSF), 50 mM NaF, 1 mM dithiothreitol (DTT) and 10 μg/ml each of leupeptin, pepstatin and aprotinin. The tissue homogenates were centrifuged at 2000 rpm in +4°C for 1 min. One quarter of the supernatant was taken for total protein extraction, one quarter for cytoplasmic and the rest of the supernatant for nuclear protein extraction. To separate the total protein fraction, 5 × NEB (100 mM Tris-HCl [pH 7.5], 750 mM NaCl, 5 mM EDTA, 5 mM EGTA, 5% Triton X100, 12 mM sodium pyrophosphate, 5 mM β-glycerophosphate, 5 mM Na_3_VO_4_) was added to the tissue homogenate followed by centrifugation at 12500 rpm for 20 min. To separate the cytoplasmic proteins, the homogenate from the first centrifugation was separated by centrifugation at 12 500 rpm for 20 min. The supernatants were frozen in liquid nitrogen and stored at -70°C until assayed.

To extract the nuclear protein fraction, the supernatant from the first centrifugation was incubated on ice for 15–30 min. NP-40 was added, and the nuclei were pelleted after centrifugation at 12 500 rpm for 30s. The resulting pellet was resuspended in a solution containing 20 mM HEPES (pH 7.9), 0.4 M NaCl, 1 mM EDTA, 1 mM EGTA, 1 mM Na_3_VO_4_, 2 mM benzamidine, 1 mM PMSF, 50 mM NaF, 1 mM DTT, 3 μg/ml 1-chloro-3-tosylamido-7-phenyl-2-butanone (TPCK), 3 μg/ml L-1-tosylamido-2-phenylethyl chloromethyl ketone (TLCK), and 10 μg/ml each of leupeptin, pepstatin and aprotinin. The samples were incubated at +4°C for 45 min and centrifuged at 12 500 rpm for 5 min. The supernatant was stored at -70°C until assayed. All protein concentrations were determined by the Bio-Rad protein assay (Bio-Rad Laboratories, CA, USA).

### Protein extraction from cultured cardiomyocytes

To extract total protein from cultured cardiomyocytes, the cells were scraped in 100–150 μl of 1 × NEB buffer containing protease-inhibitor cocktail (1:100 volume), phosphatase-inhibitor cocktail (1:100 volume) and 1 mM DTT (1:1000 volume). Cells were vigorously vortexed for 20 s and centrifuged 12500 rpm for 20 min at +4°C. The supernatant was collected as the total protein fragment. The entire procedure was carried out at +4°C. The supernatant was stored at -70°C until assayed. To extract the nuclear and cytosolic proteins from cultured NRVMs, the cells were scraped in ice-cold 1 × PBS. The cells were centrifuged at 12 500 rpm for 15 min and the pellets were resuspended in 100 μl of buffer consisting of 10 mM HEPES (pH 7.9), 10 mM KCl, 0.1 mM EDTA and 0.1 mM EGTA supplemented with protease-inhibitor cocktail (1:100), phosphatase-inhibitor cocktail (1:100 volume) and 1 mM DTT (1:1000 volume). The suspensions were allowed to swell on ice for 15 min. The cell membranes were lysed by adding 10 μl of 10% IGEPAL CA-630 detergent and vortexing vigorously for 15s followed by a centrifugation (12500 rpm for 30s). The supernatants were collected as the cytosolic fragments. The pellets were resuspended in 20 μl of buffer containing 20 mM HEPES, 0.4 M NaCl, 1 mM EDTA and 1 mM EGTA, with supplements similar to those used in buffer for resuspending cell pellets, and rocked for 15 min. The samples were centrifuged (12500 rpm for 5 min) and the supernatant was collected as the nuclear fragment. The entire procedure was carried out at +4°C. Protein concentrations were determined by colorimetric assay (Bio-Rad Laboratories).

### Western blotting

For Western blot, 15 μg of protein from cultured cardiomyocytes and 60 μg of protein from tissue samples was loaded onto a SDS-PAGE gel and transferred to nitrocellulose filters. The membranes were blocked in Odyssey Blocking Buffer–TBS (1:1) and incubated with anti-PHACTR1 polyclonal antibody (Sigma), anti-sarcoplasmic reticulum Ca^2+^-ATPase (SERCA2) (Santa Cruz, CA, USA), anti-phospholamban-Ser16, anti-phospholamban (Santa Cruz), anti-GAPDH (glyceraldehyde-3-phosphate dehydrogenase) (Millipore, MA, USA), anti-MRFT-A (myocardin-related transcriptor factor A) (Santa Cruz) or anti-cofilin phopho-Ser3 (generous gift from M.K. Vartiainen, University of Helsinki, Finland) antibody over-night. After incubation with secondary Alexa Fluor anti-rabbit, anti-mouse or anti-goat antibody (Invitrogen), the protein levels were detected using Odyssey Infrared Detection (LI-COR Biosciences, Lincoln, NE, USA).

### Histological analysis

In the histological analysis, the LVs were fixed in 10% buffered formalin solution. Transversal sections of the LV were embedded in paraffin, and 5-μm sections were cut. Sections were cut from the mid-section of the heart, at the level of the papillary muscles. Samples from different animals were obtained in an identical way and from the corresponding sites in order to make the samples fully comparable. Sections were stained with hematoxylin and eosin or Masson trichrome to examine the fibrotic area. To detect apoptotic cells, a terminal deoxynucleotidyltransferase-mediated dUTP nick end-labeling (TUNEL) assay was performed as previously described [25]. Pecam-1 (sc-1506-R, Santa Cruz Biotechnology, Santa Cruz, CA, USA) was used to stain endothelial cells. The number of capillaries was calculated from five representative high power fields (40×) from the LV of each section; 3 from epicardial and 2 from endocardial side of the LV were selected. To identify cells undergoing division, immunohistochemical labeling of nuclear Ki-67 was performed by using monoclonal mouse anti-rat Ki-67 antigen antibody (DakoCytomation, Glostrup, Denmark). The whole LV was scanned and stained cells were counted from high power fields (40×) choosing 5 hot spot areas in each sample. To examine efficiency and localization of the expression of the transgene, the sections were incubated with polyclonal anti-PHACTR1 antibody (Sigma) at a dilution of 1:500 at 3 days after gene transfer. All measurements were performed by persons blinded to the treatments.

### Gel mobility shift assay

Gel mobility shift assays were performed as previously described [19]. Briefly, double-stranded synthetic oligonucleotides containing serum response factor (SRF) binding elements (5’- ACAGGATGTCCATATTAGGACATCT -3’) of rat c-*fos* promoter [26] were labelled with α-^32^P-dCTP. Nonspecific competitor DNAs included double-stranded oligonucleotides containing mutated sites for SRF (5’- ACAGGATGTCCATATTATTACATCT -3’). Binding reactions consisted of 10 μg of nuclear protein extract. Supershift experiments were performed by preincubating the protein extract with 4 μg of SRF antibody (Santa Cruz) for 10 minutes on ice before the binding reaction. The binding reactions were carried out at room temperature for 20 minutes. Protein-DNA complexes were separated by electrophoresis on 5% polyacrylamide gel in 0.5 × TBE (Tris-borate-EDTA buffer). The gels were dried and exposed with PhosphoImager screens (Molecular Dynamics), which were scanned by a Biorad Molecular Imager (Bio-Rad Laboratories). The results were quantified by using Quantity One software (Bio-Rad Laboratories).

### Human LV Tissue Samples

LV samples were obtained from explanted human hearts from 4 patients with cardiomyopathy (2 dilated, 1 idiopathic, 1 hypertrophic) undergoing heart transplantation. Non-diseased donor hearts unsuitable for transplant were used to obtain control myocardial tissue (n = 2). All tissues were obtained with signed informed consent from the subjects. The project was approved by the local Ethics Committee of Semmelweis University, Hungary (Ethical permission number: 5012-0/2011-EKU142/PI/11–Semmelweis University, Hungary) and conducted in accordance with the guidelines of the Declaration of Helsinki.

### Relationship between MI associated PHACTR1 allele and cardiac function

The Malmö Preventive Project (MPP) started in the mid- 1970s in the Malmö University Hospital, Malmö, Sweden as a prospective population-based study [27]. Between 1974–1992, a total of 33,346 men and women from the Malmö city area were recruited and screened for conventional risk factors for all-cause mortality and cardiovascular disease. In the years 2002–2006, all survivors from the original MPP cohort were invited to a follow-up examination. Of these, 18,240 participants responded to the invitation and were re-examined [28]. There were no exclusion criteria and of those who were alive the participation rate was 72%. For the genetic studies, additional samples of peripheral venous blood were collected, as the original MPP design had not considered the possibility of conducting DNA analyses.

In a sub-sample of the MPP follow-up examination, 1792 participants underwent echocardiography on a separate visit [28]. Conventional echocardiography/Doppler and tissue Doppler imaging (TDI) were conducted with a 3V2c transducer (Acuson Sequoia, Mountain View, CA, USA) or a S3 transducer (Sonos 5500 Philips, Andover, MA, USA) [29]. Parasternal long- and short-axis, and apical four- and two-chamber views were used to evaluate cardiac dimensions and LVEF. Diastolic heart function was defined according to European Society of Cardiology guidelines and we compared the genotypic distribution of the previously MI associated PHACTR1 SNP rs12526453 [12] between subjects who had normal diastolic function or only mild diastolic dysfunction (relaxation impairment) and those with significant diastolic dysfunction (i.e. subjects displaying a pseudonormalization pattern or a restrictive pattern). Left atrial area, indexed for body surface area (BSA), was used as a continuous trait for diastolic function. Out of the 1792 subjects who underwent echocardiography, DNA was available and extracted from buffy coats with the use of QIAamp-96 spin blood kits (QIAGEN, Venlo, Netherlands) and rs12526453 was genotyped in 1550 subjects using primers and probes which were custom synthesized by Applied Biosystems (Foster City, CA, USA) according to standard recommendations for the AB Prism 7900HT analysis system, and genotyped with the polymerase chain reaction-based TaqMan method [30]. Of these 1550 subjects, diastolic dysfunction class could be determined in 1477, left atrial area in 1523 and ejection fraction in 1548.

### Statistics

The results are expressed as mean ± SEM. In the comparison between two groups, Student’s t-test was used. In the multiple comparisons, one-way analysis of variance (ANOVA) followed by least significant difference (LSD) post-hoc test was performed. A value of *P*< 0.05 was considered statistically significant.

Crude and age- and sex-adjusted logistic regression models were used to relate rs12526453 genotypes (additive model with minor allele coded) to a dichotomous dependent variable of diastolic dysfunction (presence of pseudonormalization pattern or restrictive pattern versus normal diastolic function or relaxation impairment). In crude and age- and sex-adjusted linear regression models we related rs12526453 genotypes (additive model with minor allele coded) to body surface normalized left atrial size and ejection fraction as a continuous variable.

## Results

### Rapid reduction of Phactr1 expression in experimental MI

**Fig 1:**
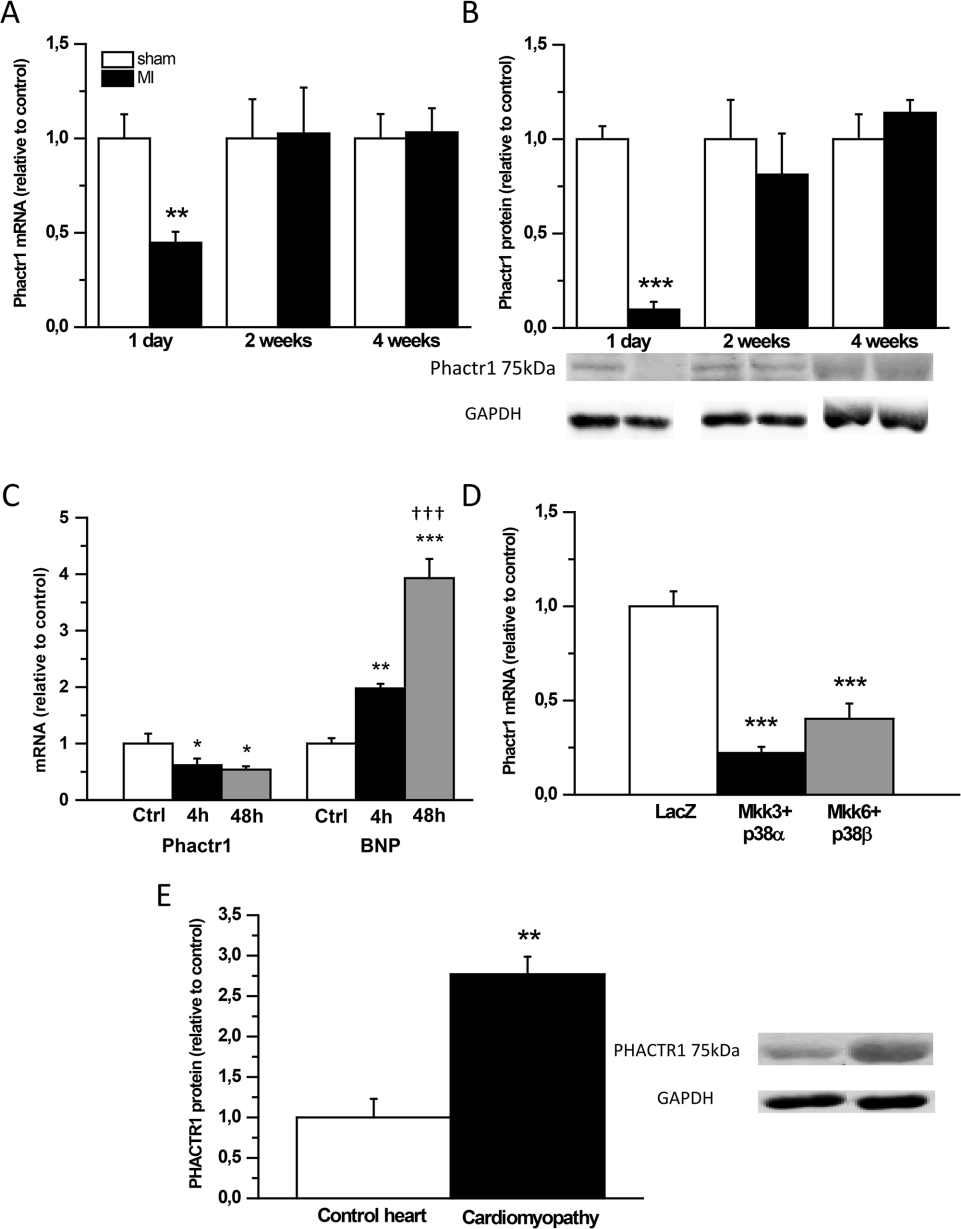
Phactr1 mRNA and protein levels are reduced 1 day after experimental myocardial infarction (MI) in rats. A) Phactr1 mRNA levels measured by RT-PCR and B) Phactr1 total protein levels analyzed by Western blot from the LV tissue samples 1 day, 2 weeks and 4 weeks after MI (n = 6–16). Open bars represent sham and solid bars MI **P<0.01, ***P<0.001 versus sham (Student’s t-test). C) Phactr1 and BNP mRNA levels in mechanically stretched neonatal ventricular myocytes *in vitro*. D) Phactr1 mRNA levels in response to p38 MAPK overexpression in neonatal ventricular myocytes *in vitro* (n = 5–9).*P<0.05, **P<0.01, ***P<0.001 versus control (Ctrl) or LacZ; ^ƗƗƗ^ *P*<0.001 versus group at 4h (one-way ANOVA followed by least significance difference post hoc test). E) PHACTR1 total protein levels assessed by Western blot analyses from human heart samples (n = 2–4). **P<0.01 versus control hearts (Student’s t-test). The results are expressed as mean±SEM. Representative Western blots are shown.

Since PHACTR1 has been associated with early-onset MI in GWAS studies [12], we first studied the effect of post-infarction myocardial remodelling on Phactr1 expression in a model of acute MI in adult rats. Both Phactr1 mRNA and protein levels were markedly reduced (60%, *P*<0.01 and 90%, *P*<0.001, respectively) at 1 day in response to MI induced by ligation of LAD in rats, and they had returned to control levels at 2 weeks (Fig 1A and 1B).

### Cyclic mechanical stretch and p38 MAPK overexpression down-regulate Phactr1 expression *in vitro*

Next, we characterized the potential mechanisms through which MI could decrease Phactr1 expression by using an *in vitro* mechanical stretch model of cultured neonatal rat ventricular myocytes (NRVMs) since stretching of cardiomyocytes occurs in the loaded heart i.e. in acute MI [31]. Mechanical stretch caused a rapid and sustained down-regulation (50–60%) of Phactr1 mRNA levels, whereas cyclic mechanical stretch increased BNP mRNA levels significantly at 4 and 48 hours (Fig 1C), as expected [32]. Furthermore, since mechanical stretch induces the MAPK cascade [33], the role of p38 MAPKs in regulating Phactr1 expression was studied in NRVMs. As shown in Fig 1D, combined overexpression of MKK3bE with both p38α and p38β isoforms was sufficient to decrease Phactr1 gene expression in cultured NRVMs.

### Increased PHACTR1 protein levels in patients with cardiomyopathy

Since GWAS studies have revealed an association with human MI, we studied whether PHACTR1 would also be expressed in the LV of control human hearts and, whether the expression levels would be altered in failing hearts. Interestingly, PHACTR1 protein levels were significantly elevated in patients with cardiomyopathy when compared to control hearts (Fig 1E). In contrast, the PHACTR1 mRNA levels did not differ between groups (data not shown).

### Augmentation of LV Phactr1 levels by adenoviral gene delivery

**Fig 2:**
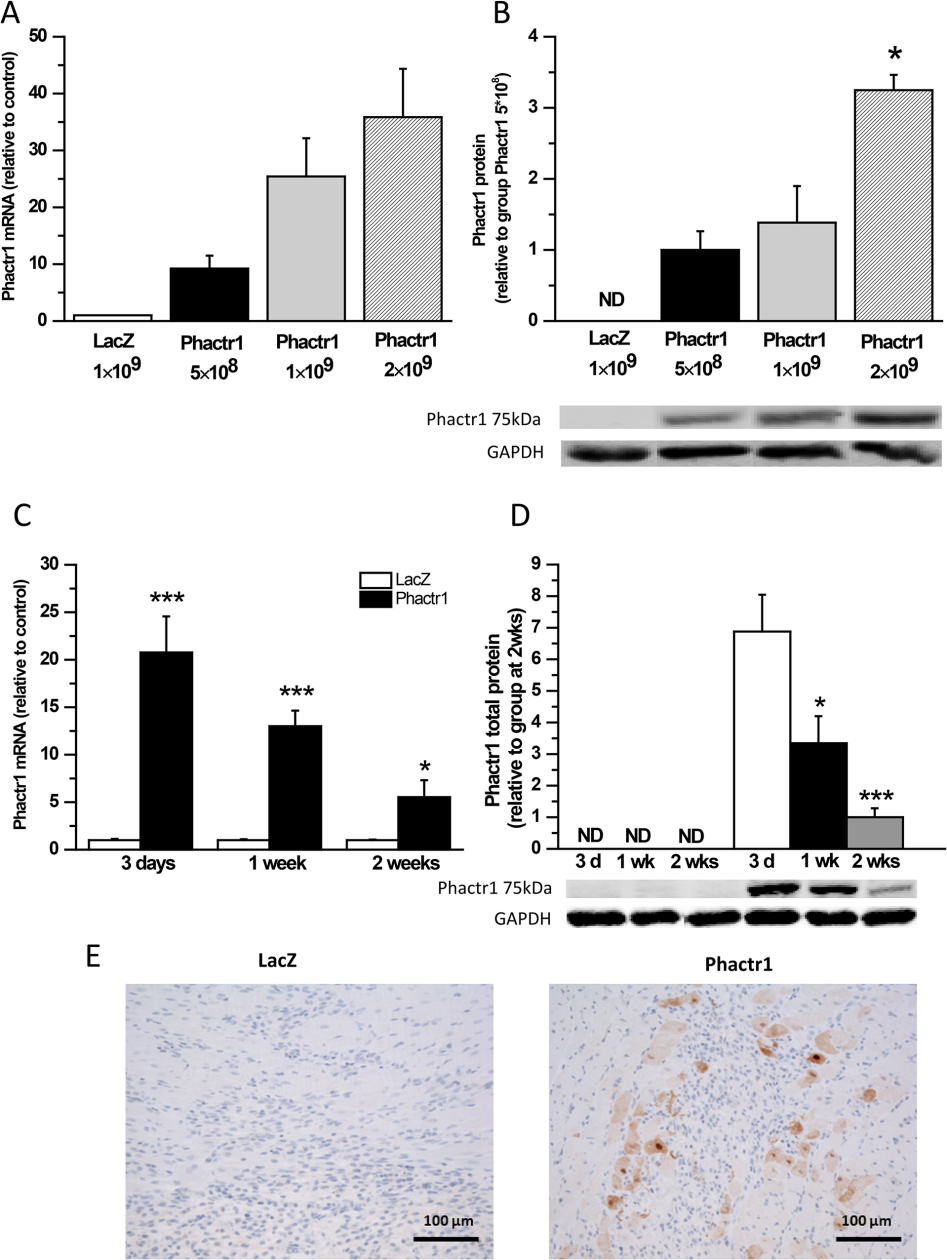
Phactr1 mRNA and protein levels increase dose-dependently after adenovirus-mediated Phactr1 gene delivery into left ventricle of rat. A) Phactr1 mRNA levels measured by RT-PCR and B) Phactr1 total protein levels analyzed by Western blot from the LV apex samples 3 days after gene delivery (n = 3). *P< 0.05 versus Phactr1 5×10^8^ (one-way ANOVA followed by least significance difference post hoc test). C) Phactr1 mRNA levels 3 days, 1 week and 2 weeks after adenoviral gene transfer. Open bars represent LacZ 5×10^8^ IFU/rat and solid bars Phactr1 5×10^8^ IFU/rat (n = 6–9). *P< 0.05, **P<0.01, ***P<0.001 versus LacZ (Student’s t-test). D) Phactr1 total protein levels 3 days, 1 week and 2 weeks after adenoviral gene transfer (n = 6–10). *P< 0.05, ***P<0.001 versus group at 2 weeks (one-way ANOVA followed by least significance difference post hoc test). ND indicates not detected. E) Efficiency of gene transfer was confirmed by immunohistochemical staining against Phactr1 at day 3 after gene transfer. The results are expressed as mean±SEM. Representative Western blots are shown.

To examine the direct myocardial effects of Phactr1, we established an *in vivo* adenoviral gene transfer protocol to restore the decreased Phactr1 levels after MI in the LV of the adult rat heart. Three different doses (5×10^8^, 1×10^9^ and 2×10^9^ infectious units) of adenoviral constructs were first tested to increase LV Phactr1 mRNA levels at day 3. Both Phactr1 mRNA and protein levels were elevated in a dose-dependent manner, with mRNA levels being nine times higher after the lowest dose (Fig 2A and 2B). Next, we studied the time-course of Phactr1 expression in response to adenovirus mediated delivery of Phactr1 into adult rat LV at a dose of 5×10^8^ infectious units, the control animals receiving virus expressing LacZ also at 5×10^8^ infectious units. Both the LV Phactr1 mRNA and protein levels were significantly elevated for up to 2 weeks (Fig 2C and 2D). When the localization of the Phactr1 expression was evaluated by immunohistochemistry, strong cytoplasmic and nuclear Phactr1 immunoreactivity localized to cardiomyocytes was observed, whereas endocardial endothelium, perivascular connective tissue and smooth muscle stained negative. No Phactr1 immunoreactivity was detected in LacZ-injected hearts (Fig 2E).

### Phactr1 regulates skeletal α-actin to cardiac α-actin switch *in vivo*

**Fig 3:**
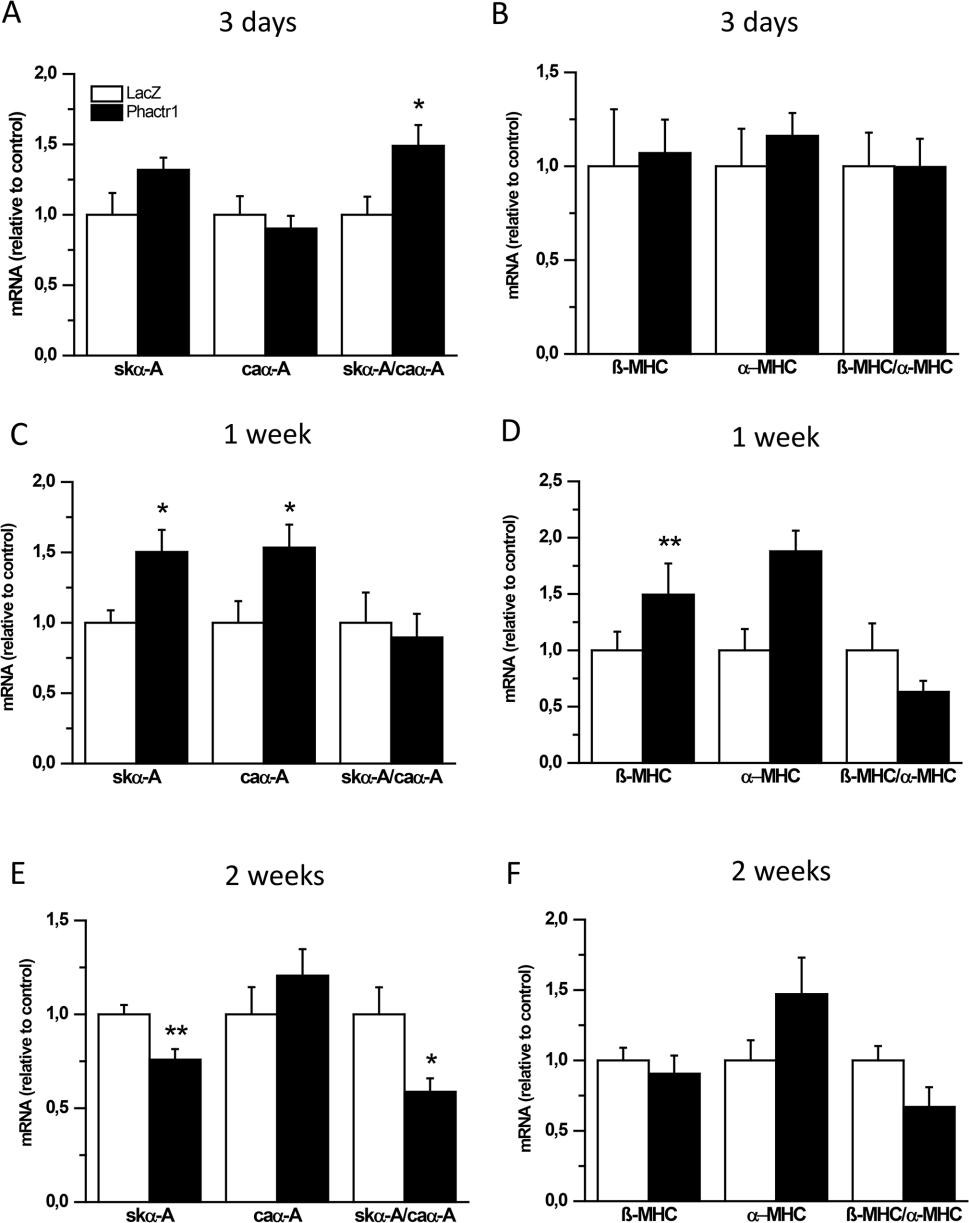
Effect of Phactr1 gene transfer on the expression of contractility protein genes in normal adult rat heart. Skeletal α-actin (skα-A), cardiac α-actin (caα-A), β-myosin heavy chain (β-MHC) and α-myosin heavy chain (α-MHC) mRNA levels were measured by RT-PCR from LV apex tissue samples 3 days (A and B), 1 week (C and D) and 2 weeks (E and F) after Phactr1 gene transfer. Open bars represent LacZ and solid bars Phactr1. The results are expressed as mean±SEM (n = 8–9).*P< 0.05, **P<0.01 versus LacZ (Student’s t-test).

**Fig 4:**
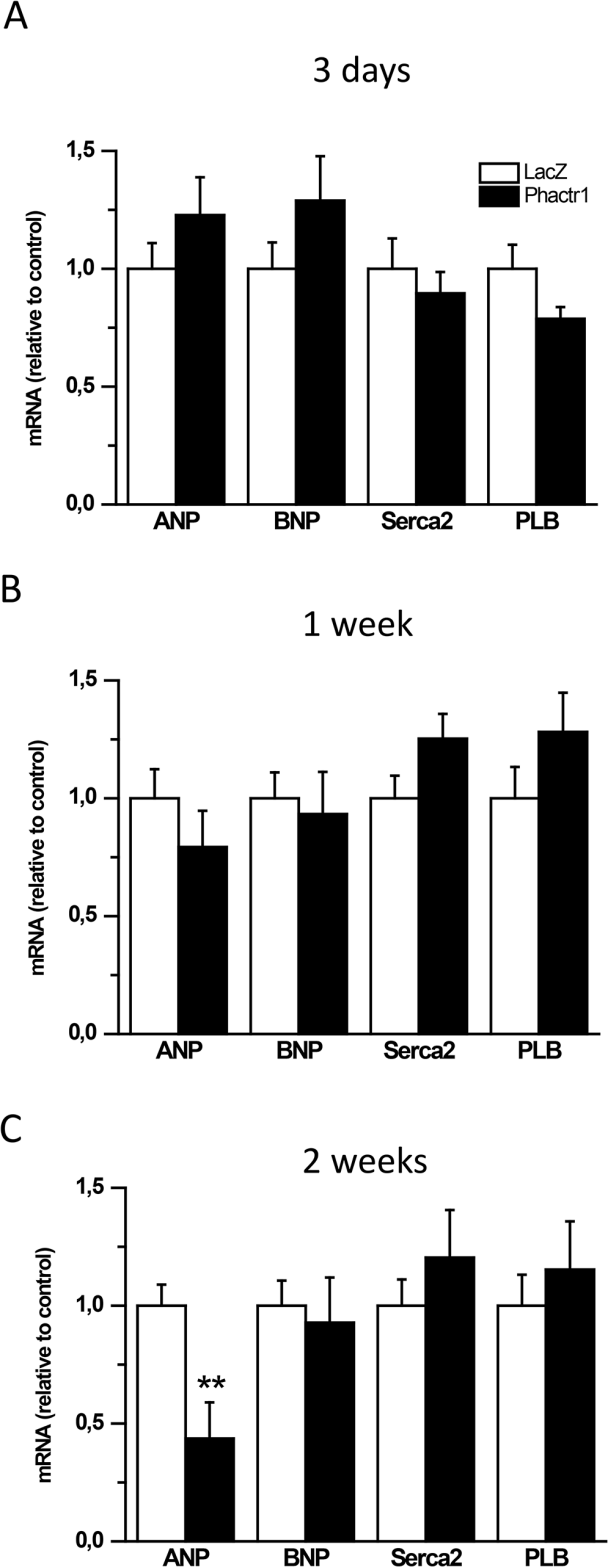
Effect of Phactr1 gene delivery on cardiac gene expression in normal adult rat heart. ANP, BNP, Serca2 and PLB mRNA levels A) 3 days, B) 1 week and C) 2 weeks after Phactr1 gene transfer into left ventricle. Open bars represent LacZ and solid bars Phactr1. The results are expressed as mean±SEM (n = 7–9). **P<0.01 versus LacZ (Student’s t-test).

Since Phactrs are a family of actin regulatory proteins and MI activates the fetal gene program, we next analyzed changes in LV contractile protein gene expression. Strikingly, Phactr1 overexpression caused the skeletal α-actin to cardiac α-actin ratio to be significantly higher (1.5-fold, *P*<0.05) 3 days after Phactr1 gene delivery but 40% lower (*P*<0.05) at 2 weeks (Fig 3A, 3C and 3E), whereas the change in β-myosin heavy chain to α-myosin heavy chain ratio was not statistically significant (Fig 3B, 3D and 3F). Furthermore, the mRNA levels for ANP, BNP, Serca2 and phospholamban (PLB) remained unchanged, with the exception that ANP mRNA levels were lower in Phactr1-treated hearts than in the LacZ-treated hearts at 2 weeks (Fig 4A through 4C).

### The effect of local myocardial Phactr1 gene delivery on cardiac structure and function

**Fig 5:**
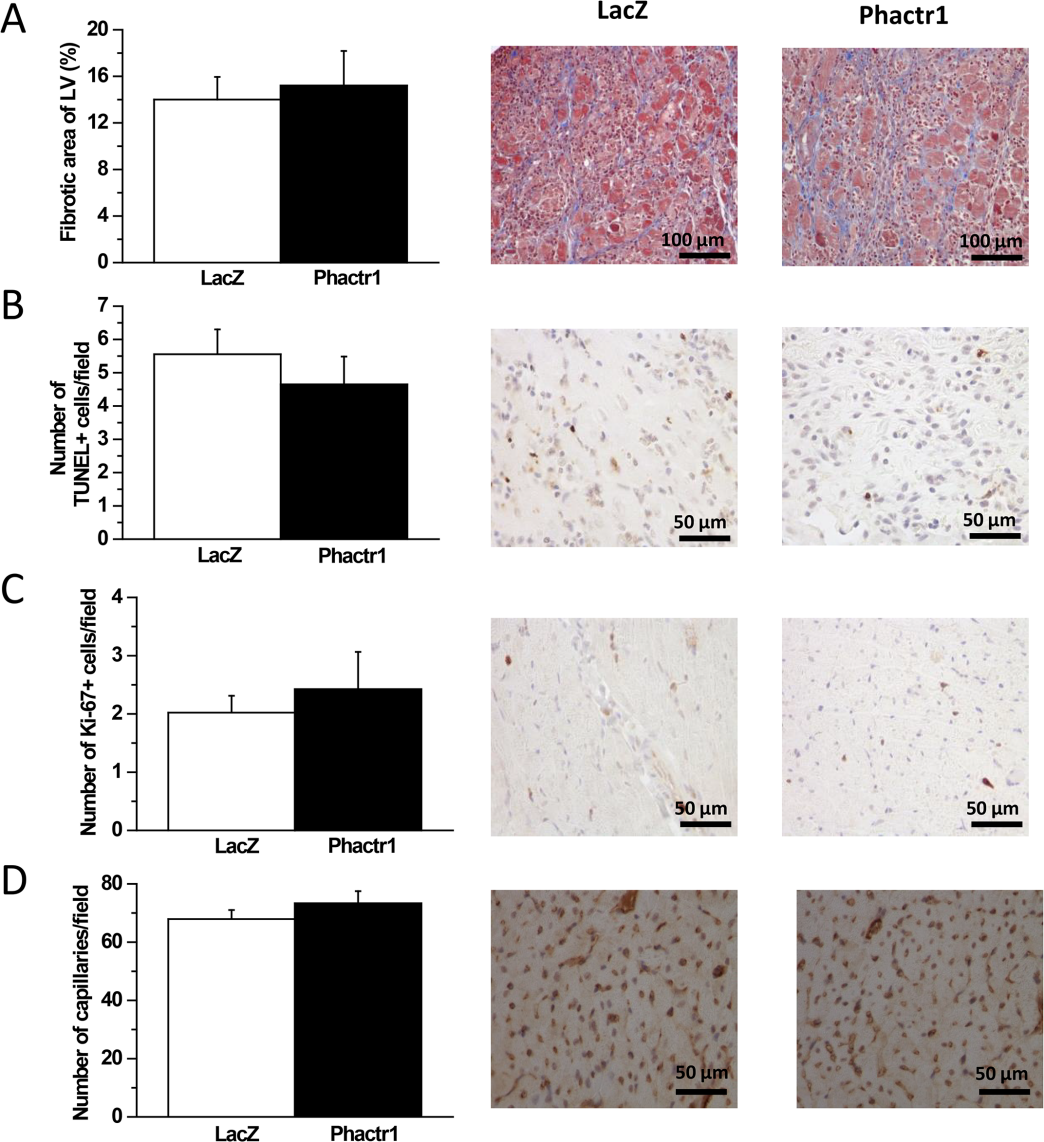
The effect of adenovirus-mediated Phactr1 gene delivery on LV fibrosis, apoptosis, proliferation and angiogenesis normal adult rats. A through D) fibrotic area, number of TUNEL+ cells, number of Ki-67+cells and number of capillaries in hearts at 2 weeks after gene transfer (n = 8–9). The results are expressed as mean±SEM.

**Fig 6:**
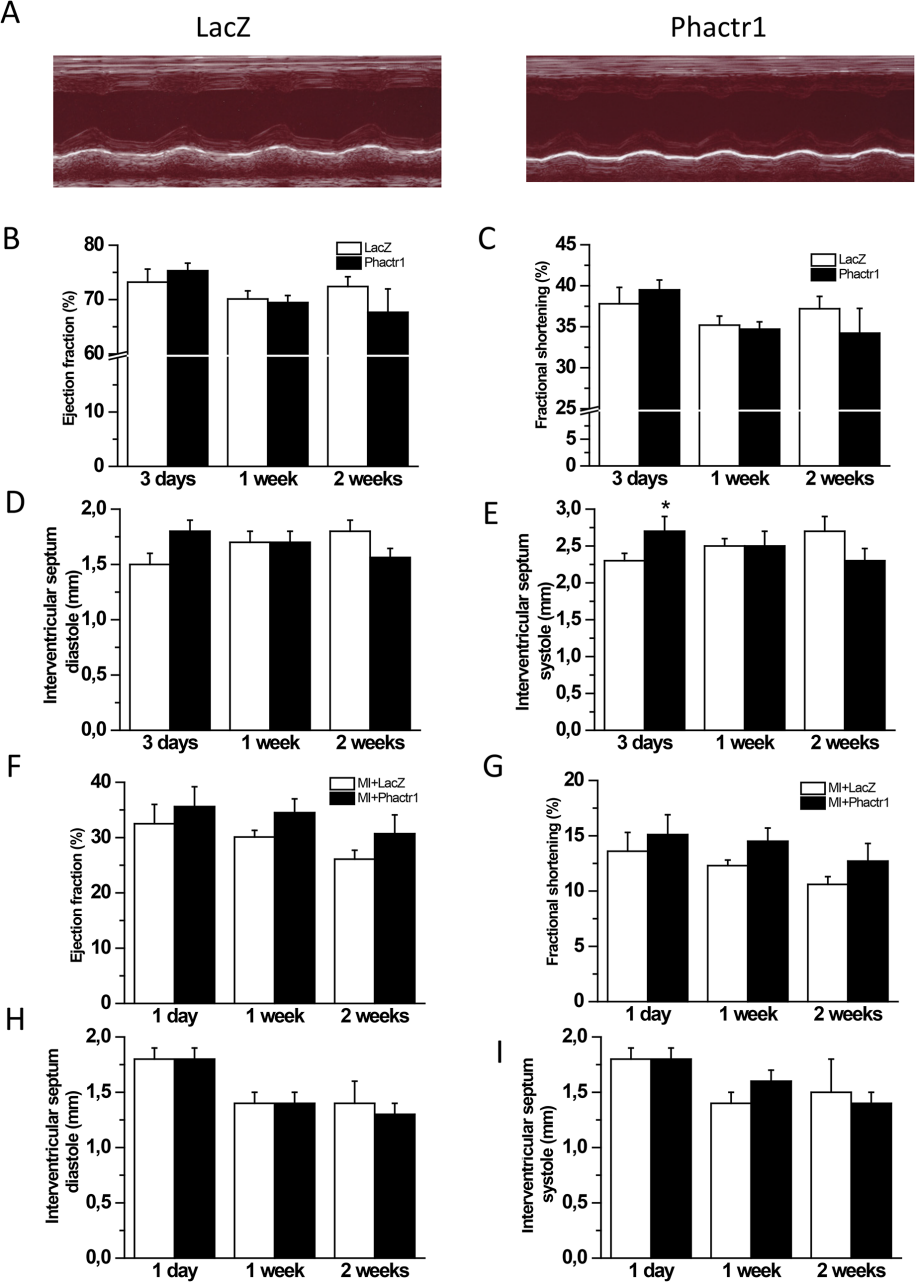
Effect of Phactr1 gene delivery on cardiac function and structure assessed by echocardiography. A) Representative M-mode images 2 weeks after Phactr1 gene transfer into normal adult rat heart. B) LV ejection fraction, C) LV fractional shortening, D) Intraventricular septum, diastole and E) Intraventricular septum, systole, after Phactr1 gene transfer into normal adult rat hearts. F) LV ejection fraction, G) LV fractional shortening, H) Intraventricular septum, diastole and I) Intraventricular septum, systole, after Phactr1 gene transfer and MI. Open bars represent LacZ or LacZ with MI and solid bars Phactr1 or Phactr1 with MI. The results are expressed as mean±SEM (n = 5–16). *P< 0.05 versus LacZ (Student’s t-test).

We next evaluated whether overexpression of Phactr1 would induce changes in cardiac structure and function. Interestingly, Phactr1 gene delivery did not have any influence on fibrosis as measured by staining histological sections with Masson’s trichrome, the number of apoptotic cells assessed by the TUNEL assay, cell proliferation (number of Ki-67 positive cells) or the number of capillaries (Fig 5A through 5D) and mean capillary area (data not shown). As assessed by echocardiography (data summarized in Table 2), there were also no statistically significant differences in LV ejection fraction (EF) or fractional shortening (FS) between LacZ- and Phactr1-treated groups (Fig 6A through 6C). In addition, the thickness of the interventricular septum (IVS) of the Phactr1-treated rats was similar to those of LacZ-treated group during diastole (Fig 6D). However, the IVS during systole was slightly but statistically significantly (*P*<0.05) thicker in the Phactr1-treated group at 3 days after Phactr1 overexpression when compared to LacZ-treated group (Fig 6E). Overall, these results indicate that Phactr1 overexpression had no major effect on LV structure and function in normal rat hearts during the 2 weeks follow-up period.

**Table 2:** Effect of intramyocardial Phactr1 gene delivery on cardiac function in adult rat heart.

Variable	Group	Phactr1	Phactr1	Phactr1	Phactr1 + AMI	Phactr1 + AMI	Phactr1 + AMI
		3 days	1 week	2 weeks	1 day	1 week	2 weeks
Interventricular septum diastole (mm)	LacZ	1.5 ± 0.1	1.7 ± 0.1	1.8 ± 0.1	1.8 ± 0.1	1.4 ± 0.1	1.4 ± 0.2
	Phactr1	1.8 ± 0.1	1.7 ± 0.1	1.6 ± 0.1	1.8 ± 0.1	1.4 ± 0.1	1.3 ± 0.1
Interventricular septum systole (mm)	LacZ	2.3 ± 0.1	2.5 ± 0.1	2.7 ± 0.2	1.8 ± 0.1	1.4 ± 0.1	1.5 ± 0.3
	Phactr1	2.7 ± 0.2*	2.5 ± 0.2	2.3 ± 0.1	1.8 ± 0.1	1.6 ± 0.1	1.4 ± 0.1
Left ventricle diastole (mm)	LacZ	7.6 ± 0.2	7.6 ± 0.3	8.1 ± 0.2	8.3 ± 0.2	9.2 ± 0.1	10.1 ± 0.2
	Phactr1	7.7 ± 0.1	7.8 ± 0.2	8.2 ± 0.1	7.9 ± 0.2	9.2 ± 0.1	10.2 ± 0.5
Left ventricle systole (mm)	LacZ	4.7 ± 0.2	5.0 ± 0.2	5.1 ± 0.2	7.2 ± 0.2	8.0 ± 0.1	9.0 ± 0.2
	Phactr1	4.7 ± 0.1	5.1 ± 0.2	5.4 ± 0.3	6.7 ± 0.2	7.9 ± 0.2	9.0 ± 0.5
Posterior wall diastole (mm)	LacZ	1.7 ± 0.1	1.7 ± 0.1	1.8 ± 0.1	2.0 ± 0.1	1.8 ± 0.1	1.7 ± 0.1
	Phactr1	1.7 ± 0.1	1.7 ± 0.1	1.6 ± 0.1	1.9 ± 0.1	1.8 ± 0.1	1.7 ± 0.1
Posterior wall systole (mm)	LacZ	2.8 ± 0.2	2.7 ± 0.1	2.7 ± 0.1	2.7 ± 0.1	2.5 ± 0.1	2.3 ± 0.2
	Phactr1	2.8 ± 0.1	2.7 ± 0.2	2.5 ± 0.1	2.5 ± 0.1	2.5 ± 0.1	2.3 ± 0.1
Ejection fraction (%)	LacZ	73.2 ± 2.4	70.1 ± 1.5	72.4 ± 1.8	32.5 ± 3.5	30.1 ± 1.2	26.1 ± 1.6
	Phactr1	75.3 ± 1.4	69.5 ± 1.3	67.7 ± 4.3	35.6 ± 3.6	34.5 ± 2.5	30.7 ± 3.4
Fractional shortening (%)	LacZ	37.8 ± 2.0	35.2 ± 1.1	37.2 ± 1.5	13.6 ± 1.7	12.3 ± 0.5	10.6 ± 0.7
	Phactr1	39.5 ± 1.2	34.7 ± 0.9	34.2 ± 3.0	15.1 ± 1.8	14.5 ± 1.2	12.7 ± 1.6
IVRT	LacZ	28.6 ± 0.5	27.8 ± 0.7	27.2 ± 0.7	21.3 ± 0.6	27.6 ± 0.8	29.0 ± 1.5
	Phactr1	28.2 ± 0.6	28.1 ± 0.7	25.5 ± 0.7	22.6 ± 0.7	27.7 ± 0.6	28.2 ± 1.3
E/A-ratio	LacZ	3.0 ± 0.4	3.3 ± 0.3	3.6 ± 0.2	3.7 ± 0.5	6.3 ± 0.4	6.1 ± 1.0
	Phactr1	3.2 ± 0.4	3.3 ± 0.4	2.8 ± 0.2*	4.5 ± 0.5	6.7 ± 0.5	5.2 ± 1.0
		n = 8–10	n = 8–9	n = 8–9	n = 11–13	n = 15–16	n = 5–7

Adenoviral gene constructs expressing Phactr1 or LacZ were injected into LV free wall with or without myocardial infarction (MI) and echocardiographic measurements were performed at 1 day, 3 days, 1 week and 2 weeks after gene transfer. The results are expressed as mean±SEM.

*P< 0.05 versus LacZ (Student’s t-test). IVRT, isovolumic relaxation time.

### Effects of Phactr1 gene delivery post-infarction

**Fig 7:**
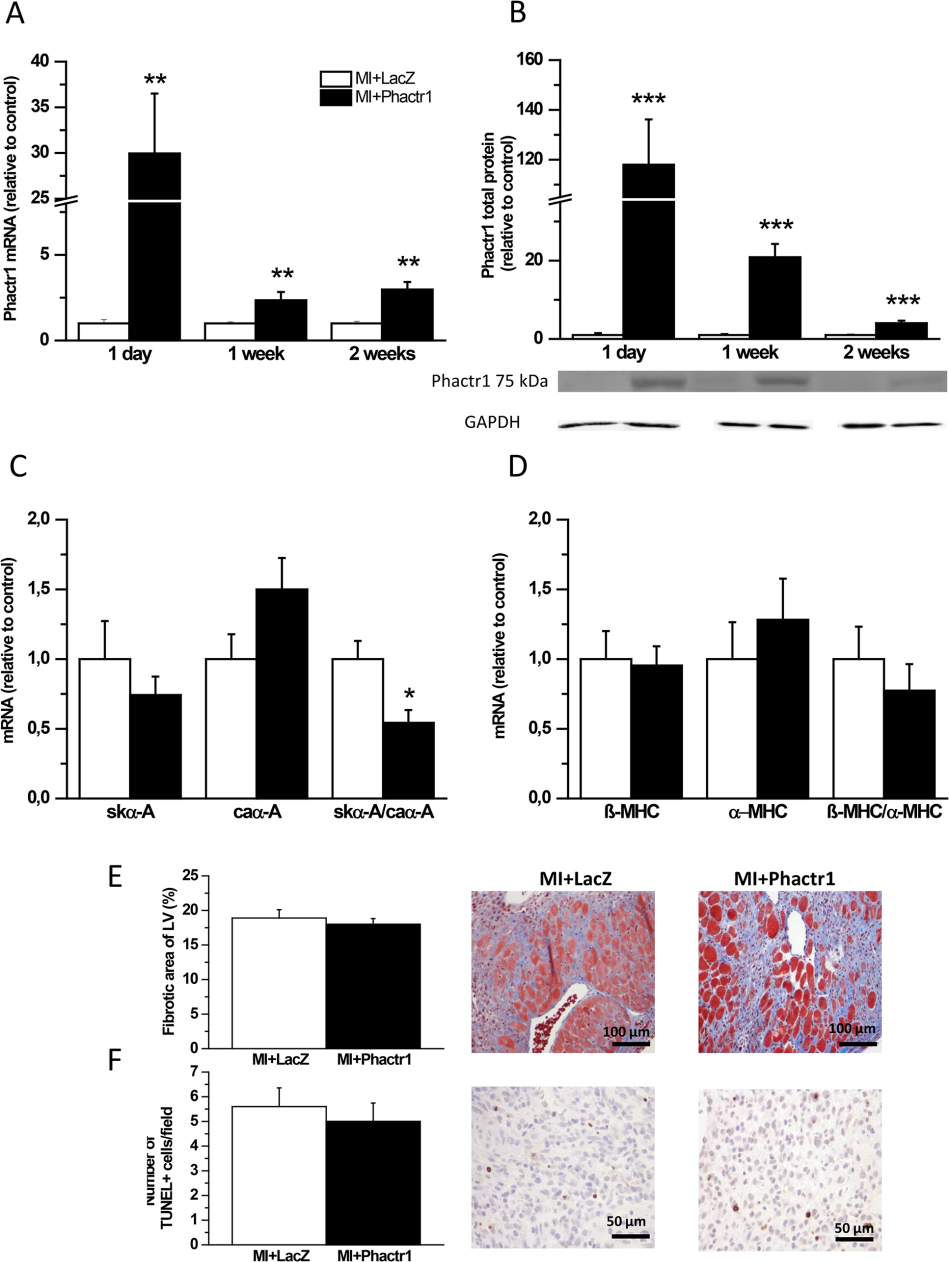
Effects of Phactr1 gene delivery post-infarction in adult rats. A) Phactr1 mRNA levels measured by RT-PCR and B) Phactr1 total protein levels analyzed by Western blot analyses from the LV tissue samples 1 day, 1 week and 2 weeks after Phactr1 gene delivery and MI. C) Skeletal α-actin (skα-A) and cardiac α-actin (caα-A) mRNA levels measured by RT-PCR and skα-A to caα-A ratio, and D) β-myosin heavy chain (β-MHC) and α-myosin heavy chain (α-MHC) mRNA levels and β-MHC to α-MHC ratio at 2 weeks after Phactr1 gene transfer and MI. Open bars represent LacZ with MI and solid bars Phactr1 with MI (n = 5–11). *P< 0.05, **P<0.01, ***P<0.001 versus LacZ with MI (Student’s t-test). E and F) Fibrotic area and number of TUNEL+ cells at 1 week after gene transfer and MI (n = 7–9). The results are expressed as mean±SEM. Representative Western blots are shown.

**Fig 8:**
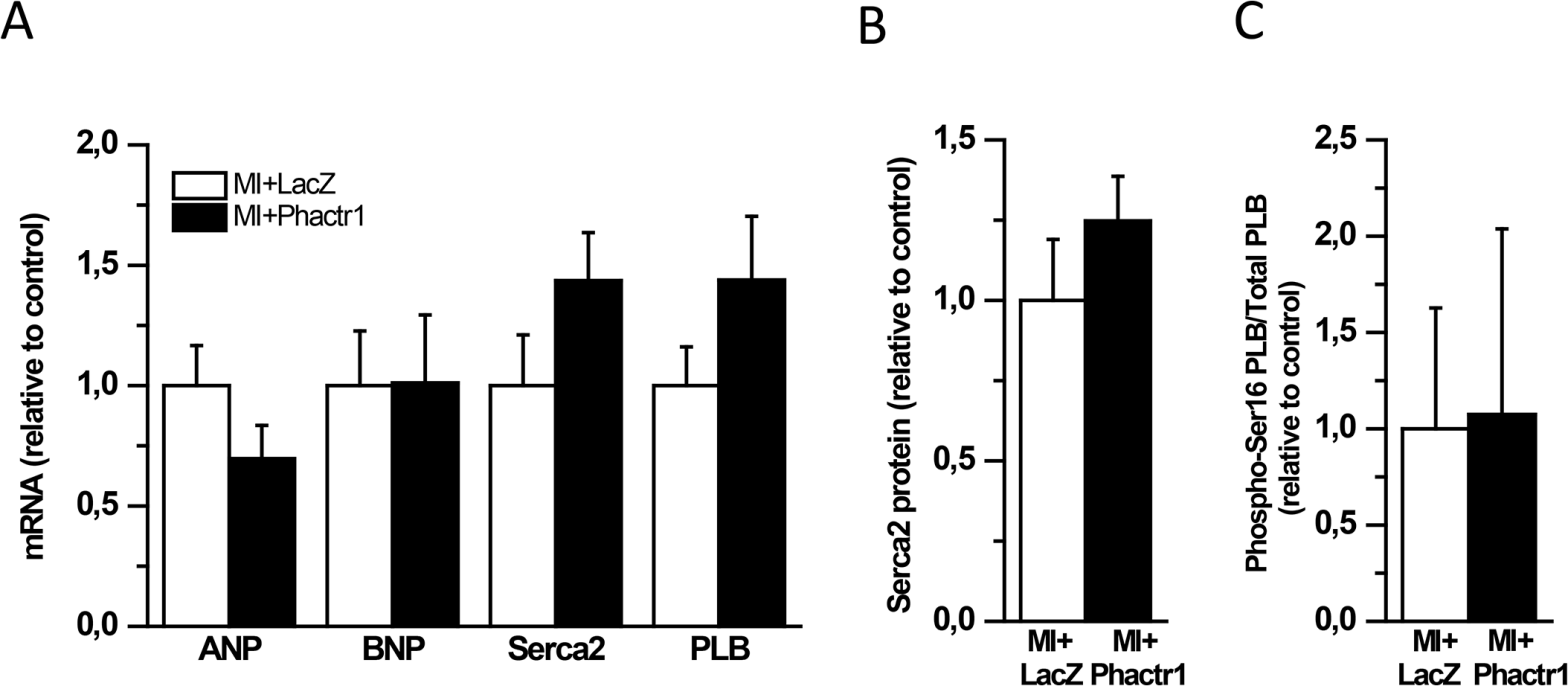
Expression of markers of cardiac hypertrophic response 2 weeks after Phactr1 gene delivery with MI. A) ANP, BNP, Serca2 and PLB mRNA levels measured by RT-PCR from LV tissue samples. B) Serca2 protein levels and C) phospho-Ser16 PLB to total phospholamban ratio analyzed by Western blot analyses from LV tissue samples. Open bars represent MI with LacZ and solid bars MI with Phactr1. The results are expressed as mean±SEM (n = 5–11).

Since Phactr1 expression was markedly down-regulated after MI, we next studied the effects of Phactr1 overexpression on cardiac gene expression, function and structure in an experimental rat MI model. RT-PCR and Western blot analysis revealed a significant increase in Phactr1 mRNA and protein levels, respectively, by adenovirus-mediated overexpression of Phactr1 up to 2 weeks after MI (Fig 7A and 7B). Consistently with the results observed in normal hearts, the skeletal to cardiac α-actin ratio was statistically significantly reduced at 2 weeks after MI (50%; *P*<0.05) (Fig 7C). Moreover, there were no statistically significant differences in the β-myosin heavy chain to α-myosin heavy chain ratio (Fig 7D) and in mRNA levels of ANP, BNP, Serca2 and PLB between LacZ- and Phactr1-treated groups (Fig 8A) 2 weeks after MI. In addition, Serca2 and PLB protein levels in Phactr1-treated group were similar to those of LacZ-treated group after MI (Fig 8B and 8C). Phactr1 gene delivery after MI did not influence the extent of fibrosis or the number of apoptotic cells (Fig 7E and 7F). There was a tendency for higher LV EF and FS in Phactr1-treated animals after MI when compared to LacZ-treated group but these changes were not statistically significant (Fig 6F and 6G). LV dimensions did not differ between Phactr1-treated hearts and LacZ-treated hearts after MI (Fig 6H and 6I).

### Phactr1 overexpression enhances SRF DNA binding activity *in vitro*

**Fig 9:**
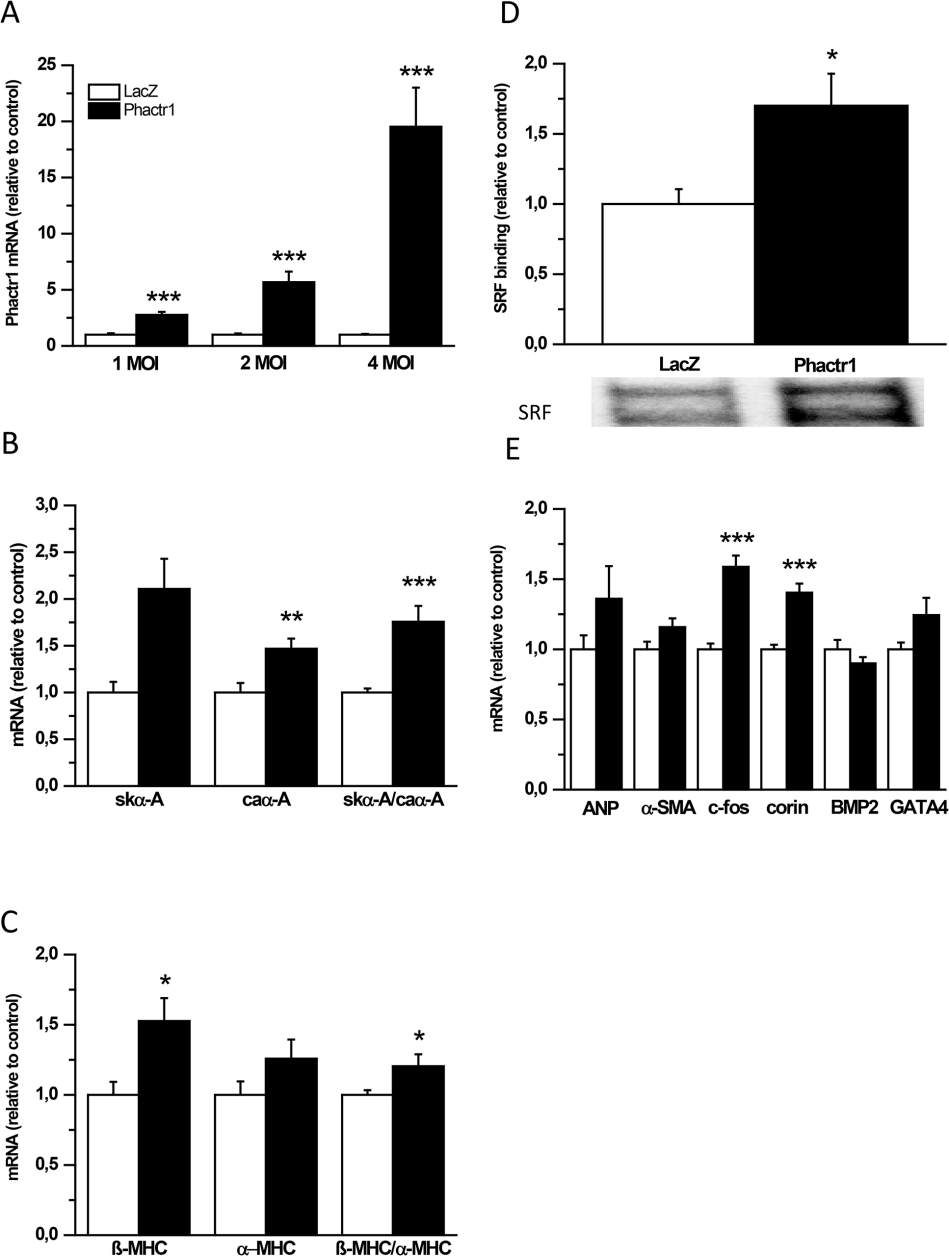
Effects of Phactr1 overexpression in cultured ventricular myocytes. A) Phactr1 mRNA levels measured by RT-PCR from cultured neonatal rat ventricular myocytes (NRVMs) transduced with Phactr1 or LacZ adenovirus for 48 hours at the virus amount of 1MOI, 2 MOI and 4 MOI. B) Skeletal α-actin (skα-A) and cardiac α-actin (caα-A) mRNA levels and skα-A to caα-A ratio, and C) β-myosin heavy chain (β-MHC) and α-myosin heavy chain (α-MHC) mRNA levels and β-MHC to α-MHC ratio from cultured NRVMs transduced with Phactr1 or LacZ adenovirus at the virus concentration of 4 MOI. Open bars represent LacZ and solid bars Phactr1 (n = 11–12 from 3 different experiments) *P<0.05, **P<0.01, ***P<0.001 versus LacZ (Student’s t-test). D) SRF DNA binding activity in cultured NRVMs transduced with Phactr1 adenovirus at the virus concentration of 1 MOI. Open bars represent LacZ and solid bars Phactr1 (n = 7–9). *P< 0.05 versus LacZ (Student’s t-test). E) ANP, α-SMA, c-fos, corin, BMP2 and GATA4 mRNA levels measured by RT-PCR from cultured NRVMs transduced with Phactr1 or LacZ adenovirus for 48 hours at the virus concentration of 4 MOI. Open bars represent LacZ and solid bars Phactr1 (n = 11–12 from 3 different experiments) ***P<0.001 versus LacZ (Student’s t-test). The results are expressed as mean±SEM.

To further evaluate the role of Phactr1 in regulating cardiac gene expression, we transfected cultured NRVMs with recombinant adenoviruses at the virus concentration of 1, 2 or 4 MOI. Phactr1 mRNA levels were elevated dose-dependently and were significantly increased already at the virus concentration of 1 MOI (Fig 9A). In agreement with *in vivo* studies at 3 days, the skeletal α-actin to cardiac α-actin ratio significantly increased in response to adenovirus-mediated Phactr1 overexpression for 48 hours used at the concentration of 4 MOI, to ensure that Phactr1 had been fully overexpressed (Fig 9B). In addition, the β-myosin heavy chain to α-myosin heavy chain ratio increased significantly in NRVMs (Fig 9C).

To examine the potential mechanisms triggering the changes in the skeletal α-actin to cardiac α-actin ratio, we measured LV serum response factor (SRF) DNA binding activity from the Phactr1 transfected cultured NRVMs by EMSA using a 28-bp oligonucleotide probe containing the SRF sites of the rat c-*fos* promoter. SRF is a member of the MADS (MCM1, Agamous and Deficiens, SRF) Box family of transcription factors that regulates a large number of cardiac muscle genes, including skeletal α-actin, cardiac α-actin and β-myosin heavy chain [34–36]. Interestingly, Phactr1 overexpression significantly increased SRF DNA binding activity in cultured NRVMs (1.7-fold; *P*<0.05) (Fig 9D). In addition, SRF-targets c-fos and corin mRNA levels increased significantly by Phactr1 overexpression, whereas other SRF-target genes ANP, α-SMA (α-smooth muscle actin), BMP2 (bone morphogenetic protein 2) and GATA4 remained unchanged (Fig 9E).The specificity of the DNA binding reactions was confirmed by performing supershift and competitor analysis (S1 Fig).

Finally, we utilized Western blot analysis to measure myocardin-related transcription factor A (MRTF-A) and cofilin-phospho-Ser3 protein levels from the Pharct1 transfected cultured NRVMs. In different muscle cell types, MRTFs including myocardin, MRTF-A and MRTF-B act as SRF binding coactivators [37], whereas Phactr4 could regulate the phosphorylation of cofilin in response to actin dynamics [38]. However, Phactr1 overexpression in cultured NRVMs exerted no significant effect on cofilin-phopho-Ser3 protein or MRTF-A levels (data not shown).

### MI associated PHACTR1 allele is not associated with cardiac dysfunction in humans

Given the findings of Phactr1 effects on the skeletal α-actin to cardiac α-actin ratio in rat heart, we hypothesized that the human Phactr1 risk allele may also affect cardiac function. The genotype frequency of the rs12526453 (C→G) was 45.2% CC, 44.1% CG and 10.7% GG. In an age and sex adjusted additive model, no significant association was detected between the rs12526453 (minor allele G coded) and the presence of significant diastolic dysfunction (pseudonormalization or restrictive pattern) (odds ratio, 95% confidence interval) (1.04, 0.86–1.26, *P* = 0.69). There was also no association between rs12526453 and left atrial area/BSA (β-coefficient±SEM) (0.12±0.08, *P* = 0.15) or with systolic function (LVEF) (0.01±0.30, *P* = 0.97).

## Discussion

Coronary artery disease and its severe complication, MI, are leading causes of mortality and morbidity all around the world [39]. Lifestyle factors are well-known risk factors for coronary artery disease and MI but genetic factors also play an important role in the disease pathogenesis. Recently several common variants associated with risk of coronary artery disease have been identified in GWAS studies [40]. Of these, PHACTR1 has been associated in early-onset myocardial infarction in GWAS irrespective of ethnic backgrounds [10–12]. However, the role of PHACTR1 in the heart is still unclear. Here we demonstrate that Phactr1 regulates the switch of skeletal and cardiac α-actin contractile protein isoforms.

The Phactr family comprises four members (Phactr1-4) which act as actin regulators and bind protein phosphatase 1 (PP1). Phactrs are highly expressed in the central nervous system and at lower levels also in heart, lung, kidney and testis [15]. Depending of the cell type, Phactrs are detected in nucleus or cytoplasm [15,38,41–43]. In agreement with this, we observed the presence of both cytoplasmic and nuclear Phactr1 immunoreactivity localized to cardiomyocytes by Phactr1 gene delivery. Phactrs contain a conserved actin binding domain called the RPEL domain which seems to regulate actin binding [15,38,42]. Indeed, Phactrs participate in cell spreading and motility [42], axon elongation [44] and regulation of actin monomer levels [38]. In some tumor cell lines, Phactr1 has been implicated in actin dynamics, cell motility and invasive behavior [17,18]. These findings indicate that Phactrs have an important role in actin dynamics.

The present study reveals that Phactr1 mRNA and protein levels are rapidly reduced after MI induced by ligation of LAD in rats suggesting that Phactr1 may have a role in the early tissue remodelling occurring post-infarction. Previously, increased PHACTR1 expression levels have been reported in HUVECs (human umbilical vein endothelial cells) after vascular endothelial growth factor (VEGF) isoform (VEGF-A_165_) treatment [45], and in human breast cancer cell line by transforming growth factor-β (TGF-β) in a miR584-dependent manner [18]. Both VEGF-A and TGF-β1 are crucial players in angiogenesis in post-infarction LV-remodelling, and PHACTR1 depletion has been associated with an impairment of tube formation and deranged endothelial cell function in HUVECs [16]. However, in our study, Phactr1 did not localize to endocardial endothelium when evaluated by immunohistochemistry nor did its overexpression have any effect on capillary density or area in normal adult rat hearts during the 2 weeks’ follow up period.

The characterization of the potential mechanisms by which MI rapidly reduced Phactr1 expression revealed that in the cyclic mechanical stretch model of cultured NRVMs, there was a marked down-regulation of Phactr1 mRNA levels. In cardiac myocytes, mechanical stretch triggers changes in gene expression via activation of intracellular signalling cascades including mitogen-activated protein kinases which are one of the best conserved mechanisms of cellular signal transduction [46,47]. Since combined overexpression of MKK3bE with both p38α and p38β gene transfer *in vitro* decreased gene expression, our results suggest that cardiac overload post-infarction down-regulates Phactr1 expression in the heart via the p38 MAPK pathway triggered by direct mechanical myocyte stretch.

Cardiac overload induced hypertrophy is known to lead to activation of skeletal α-actin expression in rat heart [48]. Normally rodent adult heart expresses the adult isoforms of contractility proteins such as cardiac α-actin and α-myosin heavy chain. In the loaded heart, however, the ratio of isoforms changes so that fetal isoforms skeletal α-actin and β-myosin heavy chain predominate [49]. Also *in vitro* in cultured cardiomyocytes mechanical loading induces the expression of skeletal α-actin [50,51]. Moreover, skeletal α-actin expression is increased in cardiac α-actin knockout mice [52]. A key finding of our present study is that Phactr1 gene delivery in the adult rat heart regulates the skeletal α-actin to cardiac α-actin ratio. The skeletal α-actin to cardiac α-actin ratio was significantly higher 3 days after Phactr1 gene delivery but lower at 2 weeks by Phactr1 overexpression. The skeletal α-actin to cardiac α-actin ratio was reduced also at 2 weeks after MI. In addition, the skeletal α-actin to cardiac α-actin ratio changed *in vitro* due to Phactr1 overexpression in cultured NRVMs. The effects of Phactr1 overexpression on cardiac gene expression were selective since Phactr1 overexpression in healthy or infarcted heart did not influence gene expression of BNP, Serca2 or PLB. However, ANP mRNA levels were reduced at 2 weeks in healthy heart and β-myosin heavy chain to α-myosin heavy chain ratio, c-fos and corin mRNA levels changed *in vitro*.

Another major finding of this study is that the change in the skeletal α-actin to cardiac α-actin ratio was associated with the increased activity of SRF. SRF is a pivotal transcriptional regulator of many of the genes necessary for cardiac function and contraction, including skeletal α-actin and cardiac α-actin [34,35]. SRF DNA binding activity was increased after Phactr1 overexpression in cultured NRVMs. SRF is expressed in many species from yeast to humans and in a wide array of cell types. SRF-dependent gene expression of different sets of target genes is triggered by SRF-binding of cell-specific cofactors which respond to distinct signals (e.g. MAPK and actin signalling) [53]. In different muscle cell types, myocardin-related transcription factors (MRTFs) including myocardin, MRTF-A and MRTF-B can act as SRF binding coactivators [37]. Cofilin is a small conserved actin-binding protein which is ubiquitously expressed in all mammalian cells [54]and it regulates actin filament dynamics and reorganization by polymerizing and/or severing actin filaments [55]. It has been reported that Phactr4 could regulate the phosphorylation of cofilin in response to actin dynamics and the overexpression of the Phactr4-humdy mutant leads to increased levels of phospho-cofilin [38]. However, in our study, Phactr1 overexpression in cultured NRVMs had no significant effect on cofilin-phopho-Ser3 protein or MRTF-A levels. It is also noteworthy, that not all SRF target genes were altered due to Phactr1 overexpression indicating that also other factors than SRF mediate the effects of overexpression on cardiac function.

Higher skeletal α-actin levels have been shown to correlate with increased contractility in BALB/c mice which due to mutation have a down-regulated cardiac isoform with a concomitant up-regulation of skeletal isoform levels [56]. Despite the role of Phactr1 as an actin regulator protein, Phactr1 overexpression had no major effect on LV structure and function in either normal or infarcted hearts during the 2 weeks’ follow-up period. There were no statistically significant changes observed in the extent of fibrosis, the number of apoptotic cells, cell proliferation, angiogenesis or in LV ejection fraction and fractional shortening between LacZ- and Phactr1-treated groups when assessed by echocardiography. However, in the present study, we could monitor functional and structural changes only during the early remodelling process, because adenoviral gene delivery results in only a transient increase in Phactr1 expression. Thus, to study the longer-term effects on cardiac function, detailed experiments in other models, such as transgenic or knockout animals, adeno-associated virus induced overexpression or gene silencing methods, will be needed. On the other hand, consistently with the results emerging from animal experiments, there was no significant association between PHACTR1 SNP rs12526453 and diastolic dysfunction in our age and sex adjusted additive model where the genotypic distribution of rs12526453 was compared between subjects having normal diastolic dysfunction or mild diastolic dysfunction and those with significant diastolic dysfunction. Moreover, the association between rs12526453 and systolic function was statistically insignificant. It is noteworthy, that PHACTR1 protein levels were up-regulated in end-stage failing hearts, and thus further studies will be required to investigate the mechanisms regulating PHACTR1 expression in human subjects in more detail.

In summary, we found that a cardiac overload induced by MI rapidly down-regulates Phactr1 expression in the left ventricles, likely due to the direct mechanical stretching of cardiac myocytes. Interestingly, Phactr1 overexpression changed the skeletal α-actin to cardiac α-actin ratio both *in vivo* and *in vitro*. Moreover, we observed enhanced SRF DNA binding activity due to Phactr1 overexpression suggesting that Phactr1 can regulate the actin isoform switch by SRF. Phactr1 overexpression did not have any significant effect on the expression of other markers of hypertrophy or on cardiac structure and function, the latter result being in agreement with the human data where no significant association has been detected between genotypic distribution of PHACTR1 locus and diastolic or systolic dysfunction. Therefore, Phactr1 acts as a regulator of the skeletal α-actin to cardiac α-actin ratio, both in healthy and in infarcted hearts.

## Supporting Information

S1 Fig Supershift and competitor analysis of SRF DNA binding activity in NRVMs. We performed supershift and competitor analysis using specific antibody and mutated DNA to confirm the specificity of the DNA-binding reactions. Each lane contained 10 μg of nuclear protein extract and double-stranded synthetic oligonucleotides containing SRF binding elements of rat c-fos promoter labelled with α-^32^P-dCTP (rc fos SRF probe). Lane 2 contained additionally a 100-fold molar excess of unlabeled rc fos SRF probe, lane 3 a 100-fold molar excess of nonspecific double-stranded oligonucleotides containing mutated sites for rc fos SRF and lane 4 SRF antibody (4 μg). SRF DNA binding activity was inhibited by a 100-fold molar excesses of unlabeled self (lane 2) but remained unaffected by mutated (lane 3). Incubation with SRF antibody resulted in an antibody-induced supershift (lane 4), indicating that the complexes bound by oligonucleotide probe contained the SRF protein.
(PDF)
