# Supplementary figures and images for: The Early-Onset Myocardial Infarction Associated PHACTR1 Gene Regulates Skeletal and Cardiac Alpha-Actin Gene Expression
Source: PLoS One. 2015 Jun 22;10(6):e0130502. doi: 10.1371/journal.pone.0130502 (PMC4476650; doi:10.1371/journal.pone.0130502)

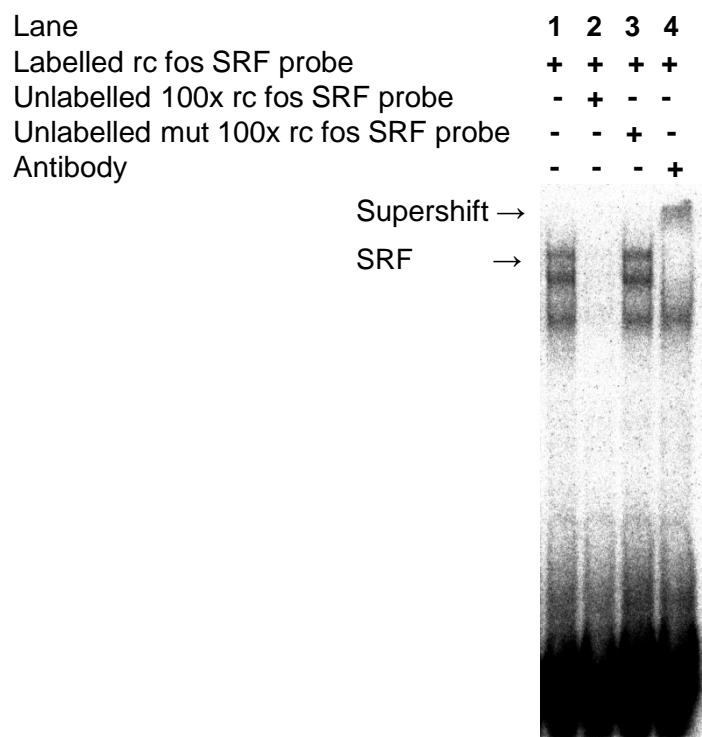

S1 Figure

Supplement: S1 Fig — We performed supershift and competitor analysis using specific antibody and mutated DNA to confirm the specificity of the DNA-binding reactions. Each lane contained 10 μg of nuclear protein extract and double-stranded synthetic oligonucleotides containing SRF binding elements of rat c-fos promoter labelled with α-32P-dCTP (rc fos SRF probe). Lane 2 contained additionally a 100-fold molar excess of unlabeled rc fos SRF probe, lane 3 a 100-fold molar excess of nonspecific double-stranded oligonucleotides containing mutated sites for rc fos SRF and lane 4 SRF antibody (4 μg). SRF DNA binding activity was inhibited by a 100-fold molar excesses of unlabeled self (lane 2) but remained unaffected by mutated (lane 3). Incubation with SRF antibody resulted in an antibody-induced supershift (lane 4), indicating that the complexes bound by oligonucleotide probe contained the SRF protein. (PDF) [file pone.0130502.s001.pdf]
